# Epigallocatechin gallate inhibits *Streptococcus pneumoniae* virulence by simultaneously targeting pneumolysin and sortase A

**DOI:** 10.1111/jcmm.13179

**Published:** 2017-04-12

**Authors:** Meng Song, Zihao Teng, Meng Li, Xiaodi Niu, Jianfeng Wang, Xuming Deng

**Affiliations:** ^1^ The First Hospital and Institute of Infection and Immunity Jilin University Changchun China; ^2^ Key Laboratory of Zoonosis Ministry of Education College of Veterinary Medicine Jilin University Changchun China

**Keywords:** *Streptococcus pneumoniae*, pneumolysin, sortase A, neuraminidases A, antivirulence, epigallocatechin gallate

## Abstract

*Streptococcus pneumoniae* (pneumococcus), the causative agent of several human diseases, possesses numerous virulence factors associated with pneumococcal infection and pathogenesis. Pneumolysin (PLY), an important virulence factor, is a member of the cholesterol‐dependent cytolysin family and has cytolytic activity. Sortase A (SrtA), another crucial pneumococcal virulence determinate, contributes greatly to the anchoring of many virulence‐associated surface proteins to the cell wall. In this study, epigallocatechin gallate (EGCG), a natural compound with little known antipneumococcal activity, was shown to directly inhibit PLY‐mediated haemolysis and cytolysis by blocking the oligomerization of PLY and simultaneously reduce the peptidase activity of SrtA. The biofilm formation, production of neuraminidase A (NanA, the pneumococcal surface protein anchored by SrtA), and bacterial adhesion to human epithelial cells (Hep2) were inhibited effectively when *S. pneumoniae* D39 was cocultured with EGCG. The results from molecular dynamics simulations and mutational analysis confirmed the interaction of EGCG with PLY and SrtA, and EGCG binds to Glu277, Tyr358, and Arg359 in PLY and Thr169, Lys171, and Phe239 in SrtA. *In vivo* studies further demonstrated that EGCG protected mice against *S. pneumoniae* pneumonia. Our results imply that EGCG is an effective inhibitor of both PLY and SrtA and that an antivirulence strategy that directly targets PLY and SrtA using EGCG is a promising therapeutic option for *S. pneumoniae* pneumonia.

## Introduction

*Streptococcus pneumoniae* (pneumococcus) is the major causative pathogen of community‐acquired pneumonia (CAP), which carries a high mortality rate as a result of acute lung injury and multi‐organ dysfunction syndrome 1. Pneumococcal infection causes various diseases, including acute otitis media, pneumonia, sepsis and meningitis, in young children, elderly people and immunocompromised individuals, and asymptomatic carriage is also common 2. In general, the attack rate of the pneumococci is very low, but frequent asymptomatic colonization results in a tremendous overall disease burden.

Pneumococcus expresses a number of well‐characterized virulence factors, including the capsule (Cps), pneumolysin (PLY), sortase A (SrtA), pneumococcal surface protein A (PspA), hyaluronidase (Hyl) and neuraminidases (NanA), which are important in the process of infections 3. The cholesterol‐dependent cytolysin PLY, an important virulence factor, is a 53‐kD protein localized to the cell wall of pneumococcus that is released upon cell lysis 4. PLY cytotoxicity, which is attributed to its cytolytic activity, is closely associated with the development of invasive pneumococcus disease. PLY alone is capable of causing the salient histological features of lobar pneumonia in rat lungs 5, and *ply*‐deficient mutants are attenuated in murine models of infection with alleviated induction of pneumocyte injury and the inflammatory response 6, 7. In addition, a novel role of PLY in enabling nasopharyngeal colonization by pneumococcus has been demonstrated recently 8. These findings indicate that PLY is an important candidate as a target for antivirulence drug development.

SrtA, another critical virulence determinant for the pathogenesis of *S. pneumoniae*, is a membrane‐localized transpeptidase found in Gram‐positive bacteria. Many surface proteins of Gram‐positive bacteria are covalently anchored to the cell wall by sortase, which recognizes a conserved carboxylic sorting motif, LPXTG (where ‘X’ is any amino acid) and catalyses a transpeptidation reaction. These surface proteins play a critical role in many processes of pathogen infection, including adherence, colonization and invasion. SrtA mutants of *Staphylococcus aureus* 9, *Listeria monocytogenes* 10, *S. pneumoniae* 11, *Streptococcus agalactiae* 12, *Streptococcus gordonii* 13 and *Streptococcus mutans* 14 exhibit significantly reduced adhesion to epithelial cells and pathogenicity in animal models. Therefore, SrtA inhibitors represent promising candidates for the development of antivirulence therapeutics against Gram‐positive bacterial infections.

NanA, the most investigated surface protein anchored by SrtA through the LPETG motif, is an enzyme that catalyses the release of terminal sialic acid residues from glycoconjugates on host cell surfaces. *Streptococcus pneumoniae* produces at least three distinct neuraminidases, of which NanA is the most active and is conserved in all strains 15. NanA is essential for pneumococcus in nasopharyngeal colonization and the development of otitis media 16 and promotes pneumococcal brain endothelial cell invasion to cause meningitis 17. Moreover, Parker *et al*. demonstrated that pneumococcal NanA is involved in biofilm formation, which contributes to the colonization process and could increase the antibiotic resistance of pneumococcus 15, 18. Several studies have suggested that inactivation of *nanA* reduces the effectiveness of pneumococcal colonization 16. Vaccination with recombinant NanA affords some protection against nasopharyngeal colonization 19, and NanA inhibition prevents pneumococcal adhesion to pulmonary epithelial cells 20.

Epigallocatechin gallate (EGCG), a major component of green tea catechins, possesses diverse biological properties, including antioxidant, anti‐inflammatory and anticarcinogenic effects 21, 22, and has some therapeutic effects for hyperglycaemia‐induced embryopathy, breast tumours and Alzheimer's disease correlated with oestrogen depletion 22, 23, 24. Zhao *et al*. 25 have demonstrated that the combination of EGCG with β‐lactams revealed a synergistic antibacterial effect on methicillin‐resistant *S. aureus* (MRSA). Other researches have shown that EGCG could neutralize staphylococcal enterotoxin B, inhibit the haemolytic activity of *L. monocytogenes* listeriolysin O and inhibit the attachment of *Streptococcus pyogenes* to human cells 26, 27, 28. However, the potential effect of this compound against pneumococcal infection has not been reported. In this study, EGCG was identified as an effective inhibitor of pneumococcal PLY and SrtA, and the inhibitory mechanisms and potential therapeutic effects of EGCG in pneumococcal infection were further investigated in a mouse model.

## Materials and methods

### Bacterial culture

The *S. pneumoniae* strain used in this study was D39 (NCTC 7466), a kind gift from Dr. David E. Briles (Department of Microbiology, University of Alabama at Birmingham).

D39 bacteria were statically cultured in Todd‐Hewitt broth with 1% yeast extract (THY media) at 37°C with 5% CO_2_. After culture overnight, the bacteria were inoculated into fresh media and grown to mid‐logarithmic growth phase (OD_600 nm_ = 0.4) for the following assays.

### Chemicals

Epigallocatechin gallate (purity > 98%), which was purchased from Chengdu Herbpurify Co., Ltd (Chengdu, Sichuan, China), was dissolved in PBS with 2% dimethyl sulfoxide (DMSO; Sigma‐Aldrich, St. Louis, MO, USA).

### Antimicrobial susceptibility test

The minimal inhibitory concentrations (MICs) of EGCG for *S. pneumoniae* were determined as described in Clinical and Laboratory Standards Institute (CLSI) document M7. Oxacillin was used as a positive control.

### Construction, expression and purification of PLY and the mutants

The DNA sequence of PLY was amplified from *S. pneumoniae* D39 genomic DNA with the primers PLY‐F and PLY‐R, digested with the endonucleases *Bam*HI and *Xho*I, and cloned into the expression vector pET‐28a to generate the pE‐PLY construct. Site‐directed mutagenesis of PLY was performed using a QuikChange site‐directed mutagenesis kit (Stratagene, La Jolla, CA, USA) to produce E277A, Y358A and R359A with pE‐PLY as the template. The primers used are shown in Table 1. The pE‐PLY and mutant constructs were transformed into *Escherichia coli* BL21 (DE3) and expressed. The soluble His‐tagged proteins were purified from the bacterial lysate by affinity chromatography using a pre‐packed His‐Trap HP column (GE Healthcare, Uppsala, Sweden) following the manufacturer's instructions. After washing off the unbound contaminating proteins, the His‐tagged proteins were eluted with elution buffer (Tris 20 mM, imidazole 300 mM, NaCl 300 mM, pH 8.0). PLY and its mutants were concentrated at 4°C using a Millipore Amicon filter (30 kD molecular weight cut‐off) for desalting and analysed by SDS‐PAGE.

**Table 1:** Primers used in this study

Primer name	Oligonucleotide (5′‐3′)^a^
PLY‐F	CGCGGATCCGCGATGGCAAATAAAGCAGTAAA
PLY‐R	CCGCTCGAGCGGCTAGTCATTTTCTACCTTAT
PLY‐E277F	CTCCTCAGACAGCGTGGAAGCAGATTTTG
PLY‐E277R	CAAAATCTGCTTCCACGCTGTCTGAGGAG
PLY‐Y358F	GACTAAGGTTACAGCTGCGAGAAACGGAGATTTAC
PLY‐Y358R	GTAAATCTCCGTTTCTCGCAGCTGTAACCTTAGTC
PLY‐R359F	GGTTACAGCTTACGCGAACGGAGATTTAC
PLY‐R359R	GTAAATCTCCGTTCGCGTAAGCTGTAACC
SrtA_ΔN81_‐F	CTGGGATCCGTTCTAACTTCTCAATGGGATG
SrtA_ΔN81_‐R	CCGCTCGAGTTAATAAAATTGTTTATATGGTTG
SrtA_ΔN81_‐T169F	GGCATGAAGATTTATCTAGCGGATAAAAATAAAG
SrtA_ΔN81_‐T169R	CTTTATTTTTATCCGCTAGATAAATCTTCATGCC
SrtA_ΔN81_‐K171F	GATTTATCTAACCGATGCCAATAAAGTTTATAC
SrtA_ΔN81_‐K171R	GTATAAACTTTATTGGCATCGGTTAGATAAATC
SrtA_ΔN81_‐F239F	GAAATCCTAACAGCTGCGAATCAACCATATAAAC
SrtA_ΔN81_‐F239R	GTTTATATGGTTGATTCGCAGCTGTTAGGATTTC

a Restriction endonuclease recognition sites or mutated codons are underlined.

### Construction, expression and purification of SrtA_ΔN81_ and the mutants

The DNA sequence of SrtA_ΔN81_ (encoding residues Val‐82 to Tyr‐247) was amplified from *S. pneumoniae* D39 genomic DNA using the primers SrtA_ΔN81_‐F and SrtA_ΔN81_‐R, digested with the endonucleases *Bam*HI and *Xho*I, and cloned into the expression vector pGEX‐6P‐1 to generate the pG‐SrtA_ΔN81_ construct. Site‐directed mutagenesis of SrtA_ΔN81_ was performed using a QuikChange site‐directed mutagenesis kit (Stratagene) to produce T169A, K171A and F239A with pG‐SrtA_ΔN81_ as the template. The primers used are shown in Table 1. The pG‐SrtA_ΔN81_ and mutant constructs were transformed into *E. coli* BL21 (DE3) and expressed. The soluble GST‐tagged proteins were purified by affinity chromatography using a pre‐packed GST GraviTrap column (GE Healthcare) following the manufacturer's instructions. After washing off the unbound contaminating proteins, the GST‐tagged proteins were digested with Precision Protease at 4°C overnight and then were eluted with SrtA buffer (Tris‐HCl 50 mM, CaCl_2_ 5 mM, NaCl 150 mM, pH 7.5). SrtA_ΔN81_ and its mutants were concentrated at 4°C using a Millipore Amicon filter (10 kD molecular weight cut‐off) for desalting and analysed by SDS‐PAGE.

### Haemolysis assay

The inhibitory effect of EGCG on PLY haemolytic activity was evaluated as per the method previously described with some modification 29. Briefly, 10 μl of purified PLY (0.4 μΜ) was added to 965 μl of PBS with a series of concentration of EGCG (0, 0.55, 1.09, 2.18 and 4.36 μΜ), vortex mixed and incubated for 20 min. at 37°C. The sample with 0 μΜ EGCG was added with the same amount of DMSO as the sample with 4.36 μΜ EGCG. Then, 25 μl of defibrinated sheep red blood cells was added, followed by incubation for 10 min. at 37°C. After centrifugation at 3000 × *g* for 5 min., the supernatant was transferred to a cuvette for OD_543 nm_ measurement. The inhibitory effects of EGCG on the haemolytic activity of PLY‐E277A, PLY‐Y358A and PLY‐R359A were also evaluated using the same method. In addition, 0.1% Triton X‐100 was used as a positive control, and PBS served as a negative control.

### Pneumolysin expression analysis by Western blot

*Streptococcus pneumoniae* D39 was grown with a series of concentrations of EGCG (0, 4.36, 8.73, 17.45, 34.9 and 69.8 μΜ). The sample with 0 μΜ EGCG was added with the same amount of DMSO as the sample with 69.8 μΜ EGCG. The bacterial pellets were suspended in SDS‐PAGE loading buffer with β‐mercaptoethanol (β‐ME) and boiled for 10 min. Equal quantities of bacterial protein were separated by 12% SDS‐PAGE and transferred onto a polyvinylidene fluoride (PVDF) membrane. The membrane was blocked, incubated with a monoclonal antibody against pneumolysin (1:1000; Abcam, Cambridge, UK), probed with HRP‐conjugated secondary antimouse antibodies (1:2000, Proteintech) and developed with ECL reagent (Thermo Scientific, Rockford, IL, USA). The protein bands were visualized using a Tanon‐4200 imager (Tanon, Shanghai, China).

### Oligomerization analysis

PLY (0.4 μM) was mixed with different concentrations of EGCG (0, 2.18, 4.36 and 8.73 μΜ). The sample with 0 μΜ EGCG was added with the same amount of DMSO as the sample with 8.73 μΜ EGCG. After incubation for 1 hr at 37°C, 5 × SDS‐PAGE loading buffer without β‐ME was added and incubated for 10 min. at 50°C. Then, 20 μl of sample was separated by 6% SDS‐PAGE and analysed by Western bolt.

### Sortase activity inhibition assay

The inhibitory effect of EGCG against SrtA was evaluated using a fluorescence resonance energy transfer (FRET) assay involving the cleavage of the fluorescent synthetic peptide substrate Dabcyl‐QALPETGEE‐Edans (GL Biochem, Shanghai, China) as per the method previously described with some modifications 30, 31. In brief, the assay involves two reactions that were performed in a black 96‐well plate. First, 90‐μl aliquots of SrtA buffer containing 5 μΜ SrtA_ΔN81_ and various concentrations of EGCG were prepared and incubated for 30 min. at 37°C. Second, 10 μl of Dabcyl‐QALPETGEE‐Edans (10 μM) was added, and the reactions were continued for 1 hr at 37°C. Finally, the fluorescence in the reaction solution was analysed with a microplate reader (TECAN, Grodig, Austria) at an excitation wavelength of 350 nm and an emission wavelength of 520 nm. The remaining enzyme activity (%) was calculated according to the following formula: [(S – S_0_)/(C – C_0_)] × 100, where C is the fluorescence of the control (Proteinase K, SrtA buffer and substrate) after incubation for 1 hr, C_0_ is the fluorescence of the control before incubation, S is the fluorescence of the tested samples (SrtA_ΔN81_, EGCG, SrtA buffer and substrate) after incubation for 1 hr, and S_0_ is the fluorescence of the tested samples before the incubation. The inhibitory effects of EGCG against SrtA_ΔN81_‐T169A, SrtA_ΔN81_‐K171A and SrtA_ΔN81_‐F239A were also evaluated using the same method. Additionally, proteinase K was used as a positive control, and SrtA buffer served as a negative control.

### NanA and SrtA production analysis by Western blot

NanA and SrtA were detected among the total cell‐associated proteins of *S. pneumoniae* D39 as per the method previously described 11, 32. *Streptococcus pneumoniae* D39 was statically cultured with different concentrations of EGCG (0, 8.73, 17.45 and 34.9 μΜ) for 5 hrs at 37°C with 5% CO_2_. The sample with 0 μΜ EGCG was added with the same amount of DMSO as the sample with 34.9 μΜ EGCG. Upon reaching mid‐logarithmic growth phase (OD_600 nm_ = 0.4), the bacteria were diluted 1:100 in fresh medium with the corresponding concentrations of EGCG (0, 8.73, 17.45 and 34.9 μΜ), and the culture was continued for 8 hrs. Then, the 3‐ml cultures were centrifuged, and the bacterial pellets were re‐suspended in 80 μl of lysozyme buffer (20 mM Tris‐Cl, 20 mg/ml lysozyme buffer, pH 8.0) and incubated for 1 hr at 37°C. After incubation, 20 μl of 5 × SDS‐PAGE loading buffer with β‐ME was added and boiled for 20 min. A 20‐μl aliquot of bacterial proteins was separated by 10% SDS‐PAGE and analysed by western bolt with murine anti‐NanA and anti‐SrtA serum as the primary antibodies.

### Biofilm formation

As per the method previously described with some modifications 33, 34, *S. pneumoniae* D39 grown to mid‐logarithmic phase was diluted 1:100 with fresh sterile THY medium, and 1‐ml aliquots were added in triplicate to the wells of a 24‐well, flat‐bottom, polystyrene microtitre plate with different concentrations of EGCG (0, 8.73, 17.45, 34.9 and 69.8 μΜ) and incubated statically for 18 hrs at 37°C with 5% CO_2_. The sample with 0 μΜ EGCG was added with the same amount of DMSO as the sample with 69.8 μΜ EGCG. After incubation, the medium was removed by pipetting, and the plates were gently washed three times with sterile PBS. The plates were air‐dried, followed by staining with 400 μl of 0.1% crystal violet for 15 min. Excess stain was decanted off, and the plates were washed three times with sterile distilled water. The plates were allowed to dry and were then photographed. Then, the bound crystal violet was dissolved in 200 μl of 33% acetic acid, and the OD_570_ was measured using a microplate reader. The sample without EGCG and DMSO was used as a positive control, and the THY medium served as a negative control.

In addition, the biofilm biomass was calculated as follows. Pneumococcal biofilms were formed and washed as described above. The biofilm bacteria detached with trypLE Express (Invitrogen, Carlsbad, CA, USA) were serially diluted with sterile water and plated on blood agar plates for the enumeration of colony forming units (CFU). The biofilm biomass grown without EGCG was set as 100% for statistical analysis.

### Live/dead and cytotoxicity assays

Human lung epithelial cells (A549, ATCC) were cultured using Dulbecco's Modified Eagle Medium (DMEM) (Invitrogen) supplemented with 10% foetal bovine serum (Invitrogen). As per the method previously described 35, A549 cells were seeded into 96‐well plates at 2 × 10^4^ cells per well and incubated with PLY‐WT, PLY‐E277A, PLY‐Y358A or PLY‐R359A (80 nM) that had been pre‐incubated with various concentrations of EGCG (0, 1.09, 2.18, 4.36, and 8.73 μΜ) for 20 min. at 37°C. The sample with 0 μΜ EGCG was added with the same amount of DMSO as the sample with 8.73 μΜ EGCG. After incubation for 5 hrs at 37°C, cell viability was determined by measuring lactate dehydrogenase (LDH) release using a Cytotoxicity Detection Kit (LDH) (Roche, Basel, Switzerland) following the manufacturer's instructions. The sample treated with 0.02% Triton X‐100 was used as a positive control, and untreated sample served as a negative control. In addition, cells were stained with a live/dead (green/red) reagent (Invitrogen) and photographed with a confocal laser scanning microscope (Olympus, Tokyo, Japan).

### Adherence to human epithelial cells

Human larynx carcinoma epithelial cells (Hep2; ATCC CCL‐23) were cultured in complete medium containing DMEM and 10% foetal bovine serum at 37°C with 5% CO_2_.

Hep2 cells were seeded into 24‐well plates with 1 × 10^5^ cells per well in complete medium and grown to 80% confluence (12 hrs). To assess the adherence of *S. pneumoniae* to Hep2 cells, D39 cells grown to mid‐logarithmic phase with different concentrations of EGCG (0, 8.73, 17.45, 34.9 and 69.8 μΜ) were collected, washed once with DMEM, resuspended in DMEM with the corresponding concentrations of EGCG (0, 8.73, 17.45, 34.9 and 69.8 μΜ), and added to the confluent monolayers (multiplicity of infection, 30) as previous described 3, 11. The sample with 0 μΜ EGCG was added with the same amount of DMSO as the sample with 69.8 μΜ EGCG. After incubation for 2 hrs at 37°C with 5% CO_2_, the culture fluid was removed from each well, and the cells were washed three times with PBS (pH 7.4). Then, the Hep2 cells were detached from the plates by treatment with 200 μl of 0.25% trypsin (containing 0.02% EDTA) and lysed by adding 800 μl of 0.02% Triton X‐100. The numbers of D39 cells adherent to Hep2 cells were calculated by the serial dilution and plating method and compared to the initial inoculum. The sample without EGCG and DMSO was used as a positive control, and DMEM served as a negative control.

### Mouse model of intranasal lung infection

Eight‐week‐old female BALB/c mice weighing 20 ± 2 g were purchased from the Experimental Animal Centre of Jilin University (Changchun, Jilin, China). Animal experiments were approved by and conducted in accordance with the guidelines of the Animal Care and Use Committee of Jilin University.

*Streptococcus pneumoniae* D39 was grown to mid‐logarithmic phase (OD_600 nm_ = 0.4) in THY medium at 37°C and centrifuged. After washing three times with PBS, the bacteria were resuspended in PBS. For lung infection and wet/dry weight ratio study, mice were lightly anaesthetized by inhalation of ether and inoculated with 1.5 × 10^8^ CFU of pneumococci in the left naris. For the survival experiments, mice were inoculated with 2 × 10^8^ CFU of pneumococci. Each experimental group contained 10 mice. To investigate the effect of EGCG treatment, mice were subcutaneously administered EGCG (50 mg/kg) after infection and then at 8‐hrs intervals. The control mice were treated with PBS (containing 2% DMSO) on the same schedule. The mice were killed with anaesthesia followed by cervical dislocation at 48 hrs post‐infection. The lungs were weighed and homogenized for calculation of the bacterial burden *via* the serial dilution and plating method. For histopathological analysis, the lungs were placed in 10% formalin, followed by staining with haematoxylin and eosin and visualization by light microscopy 36. For the lung wet/dry weight ratio analysis, the left lung was isolated and the wet weight was measured. After the lung tissue was dried for 72 hrs at 70°C, the dry weight was measured. Then the wet/dry weight ratio of lung was calculated.

### Molecular modelling

In this work, the initial structures of SrtA and PLY were obtained from the 3D X‐ray structures (PDB code: 4O8L and 4QQA). The starting structure of the ligand/protein complex for molecular dynamics (MD) simulation was obtained based on the standard docking procedure for a rigid protein and a flexible ligand with AutoDock 4 37, 38. Then, the MD simulation was conducted for the complexed systems; the detailed processes of the computational biology method were described in previous reports 39, 40.

### Statistical analysis

The experimental data were analysed with SPSS 13.0 (Chicago, IL, USA) statistical software. An independent Student's *t*‐test was used to determine statistical significance, and *P *<* *0.05 was considered statistically significant.

## Results

### EGCG inhibits the haemolytic activity of PLY

**Figure 1:**
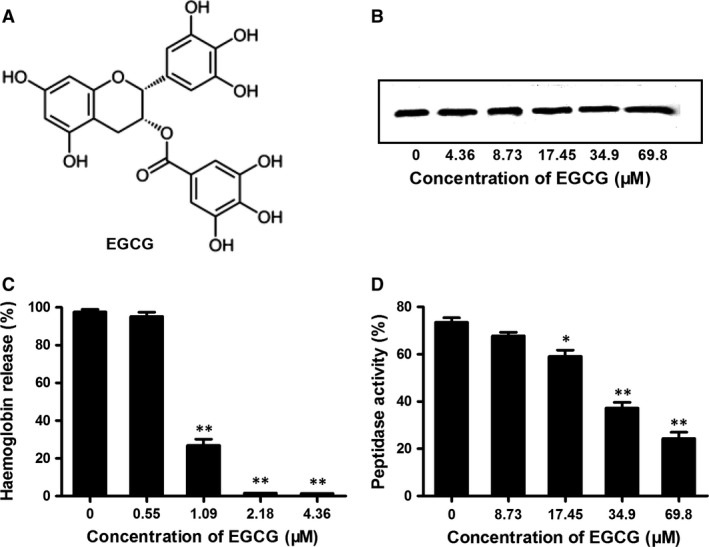
EGCG inhibits PLY haemolytic activity and SrtA_ΔN81_ peptidase activity. (**A**) Chemical structure of EGCG. (**B**) Western blot analysis of PLY expression in culture precipitates of *Streptococcus pneumoniae* D39 treated with the indicated concentrations of EGCG. (**C**) Haemolysis assays were performed with purified PLY‐WT in the presence of various concentrations of EGCG using rabbit red blood cells in PBS. (**D**) The inhibition of SrtA_ΔN81_ peptidase activity by EGCG. Purified SrtA_ΔN81_‐WT and various concentrations of EGCG were incubated for 30 min. at 37°C, followed by the addition of SrtA_ΔN81_ substrates and the fluorescent peptide Dabcyl‐QALPETGEE‐Edans and incubation for 1 hr. Finally, the fluorescence values of the reaction system were measured (excitation and emission wavelengths of 350 and 520 nm, respectively). The bars show the mean values of three independent assays. The error bars indicate the standard deviations (S.D.). * indicates *P* < 0.05 and ** indicates *P* < 0.01 compared with the drug‐free group, according to 2‐tailed Student's *t*‐tests. EGCG: epigallocatechin gallate, PLY: pneumolysin, SrtA: sortase A.

Epigallocatechin gallate (Fig. 1A), the most abundant catechin in tea polyphenols, has been widely investigated for its potential therapeutic effects in a broad range of diseases, including hyperglycaemia‐induced embryopathy, breast tumours and Alzheimer's disease correlated with oestrogen depletion 22, 23, 24. In the present study, EGCG exhibited little anti‐*S. pneumoniae* activity, with an MIC > 2234 μM. However, the haemolytic activity of purified PLY was significantly inhibited following pre‐incubation with EGCG in a dose‐dependent manner (Fig. 1C). Many natural compounds reduce bacterial haemolytic activity by inhibiting the expression of hemolysin or by directly neutralizing the activity of hemolysin 36, 41, 42. In this study, incubation of EGCG at concentrations sufficient for haemolysis inhibition did not detectably affect the expression of PLY (Fig. 1B). Thus, EGCG directly interacted with PLY and neutralized its pore‐forming activity without affecting PLY expression.

### EGCG inhibits the peptidase activity of SrtA

Fluorescence of the Edans fluorophore within the Dabcyl‐QALPETGEE‐Edans peptide is quenched by the close proximity of Dabcyl. When the peptide is cleaved, the Edans and Dabcyl fluorophores are separated, and the fluorescence increases. The inhibitory effect of EGCG on SrtA was determined by the FRET assay. When purified SrtA_ΔN81_ was incubated with the substrate peptide, increased fluorescence was observed. However, the peptidase activity of SrtA_ΔN81_ was significantly inhibited following pre‐incubation with EGCG in a dose‐dependent manner (Fig. 1D). Thus, simultaneous inhibition of PLY and SrtA activity by EGCG was observed under our experimental conditions.

### EGCG inhibits the oligomerization of PLY

The cytolytic activity of PLY, which is crucial for the virulence of *S. pneumoniae*, proceeds *via* the oligomerization of soluble monomers to form a pre‐pore complex on the target cell membrane. Blockage of the oligomerization process would remarkably inhibit the cytolytic activity of PLY.

**Figure 2:**
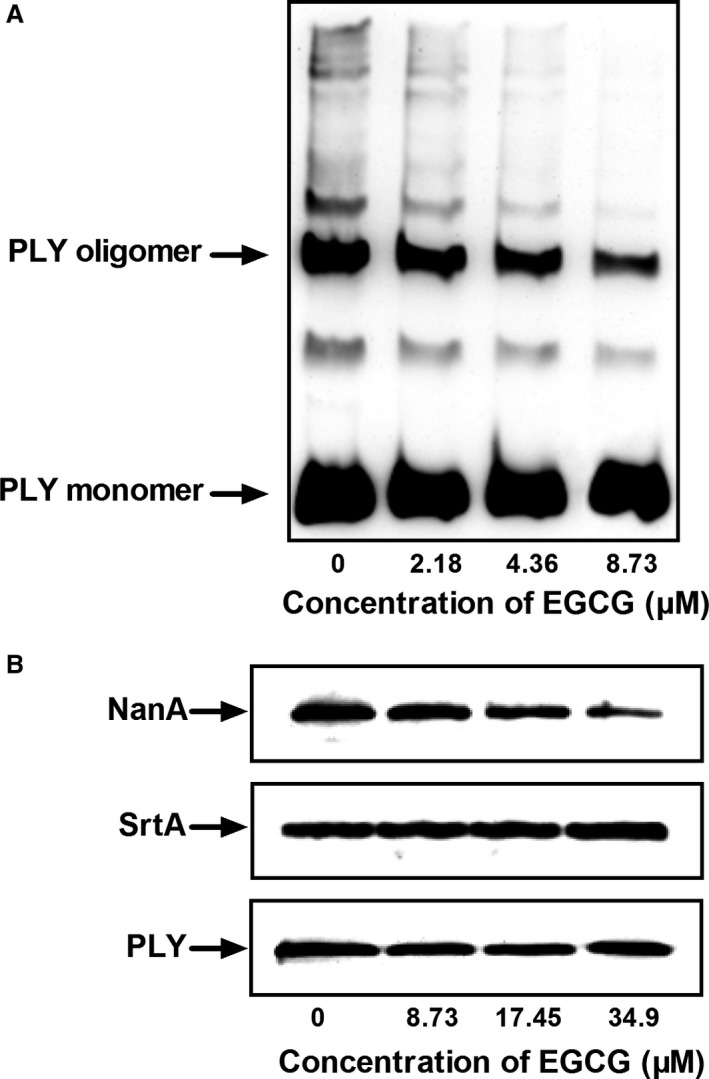
EGCG inhibits the oligomerization of PLY and the localization of NanA. (**A**) The inhibitory effect of EGCG on PLY oligomerization. Purified PLY‐WT was incubated with 4.36 and 8.73 μM EGCG, and the oligomerization of PLY was analysed by Western blot. (**B**) The influence of EGCG on NanA localization and SrtA expression in *Streptococcus pneumoniae*. D39 cells were cocultured with the indicated concentrations of EGCG and centrifuged, and the cell‐associated proteins were analysed by Western blot with murine anti‐NanA and anti‐SrtA serum. PLY was used as the loading control and detected with a monoclonal antibody. NanA: neuraminidase A. EGCG: epigallocatechin gallate.

We thus determined whether the attenuation of the cytolytic effect of PLY was due to an inhibitory effect of EGCG on PLY oligomerization. The mechanism of action of EGCG was then verified using an oligomerization assay. PLY monomers in solutions lacking cholesterol or membranes can self‐associate to form oligomers 43. The assay confirmed that PLY in the control group formed oligomers by self‐association. However, oligomerization was significantly reduced by coincubation of PLY with EGCG, in a concentration‐dependent manner (Fig. 2A). Consequently, EGCG attenuates the cytolytic activity of PLY by inhibiting its oligomerization.

### EGCG attenuates the production of NanA on the cell wall

The C‐terminal region of NanA contains the LPETG motif through which NanA is anchored to the cell surface by SrtA. All *S. pneumoniae* clinical isolates express surface‐anchored NanA, which is essential for the successful colonization and infection of the upper respiratory tract 44 and for brain endothelial cell invasion 17. Kharat *et al*. 11 demonstrated that the inactivation of the *srtA* gene in *S. pneumoniae* causes the release of most of the NanA into the growth medium. To confirm that EGCG interferes with the production of NanA on the cell wall, we determined the amounts of NanA in cell‐associated proteins of D39 pellets by Western blotting assay. The expression of PLY, which was not affected by EGCG, was used as the loading control for the samples. We observed that NanA was clearly decreased after the coculture of D39 cells with EGCG, whereas SrtA expression was not influenced (Fig. 2B).

### Identification of the binding mode of EGCG with PLY and SrtA_ΔN81_

**Figure 3:**
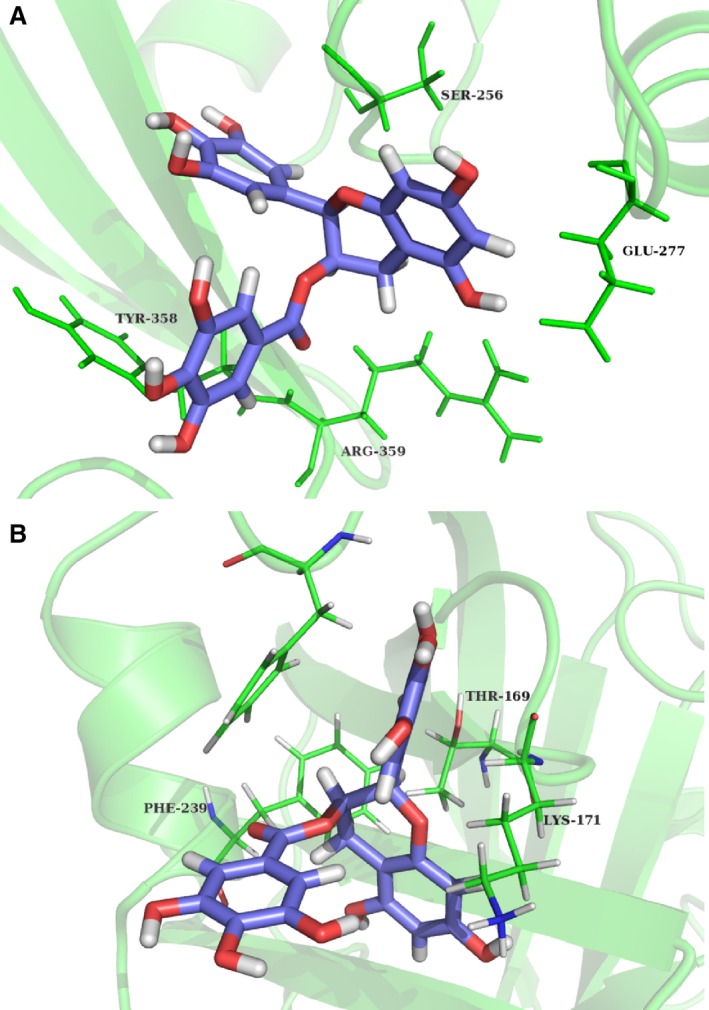
The binding modes of PLY‐EGCG (**A**) and SrtA‐EGCG (**B**). The binding sites of EGCG with PLY (Ser256, Glu277, Tyr358 and Arg359) or SrtA (Thr167, Lys169 and Phe237) are identified. EGCG: epigallocatechin gallate.

The preferential binding mode of PLY with EGCG was determined by 200‐ns molecular dynamics simulations based on the docking results. As shown in Figure 3A, the complex reached equilibrium at 100 ns based on the analysis of the root‐mean‐square deviations (RMSD) of backbone C_α_ atoms. EGCG can bind to the cleft between domains three and four in PLY *via* hydrogen bonding and hydrophobic interactions. This cleft is reported to participate in reactivity and is important for PLY 29, 35. In detail, the binding model of EGCG with PLY revealed that the side chain of EGCG can form strong interactions with Ser256, Glu277, Tyr358 and Arg359, respectively. The interaction between EGCG and SrtA was also explored by theoretical chemistry using the same method. As shown in Figure 3B, EGCG can bind to the ‘activity’ region of SrtA *via* hydrogen bonding and hydrophobic interactions. This region is reported to participate in reactivity and is important for SrtA 45, 46. In detail, the binding model of EGCG with SrtA revealed that the side chain of EGCG forms strong interactions with Thr169, Lys171 and Phe239, respectively. The above results confirm that EGCG can inhibit toxin activity by direct interaction with PLY and SrtA.

**Figure 4:**
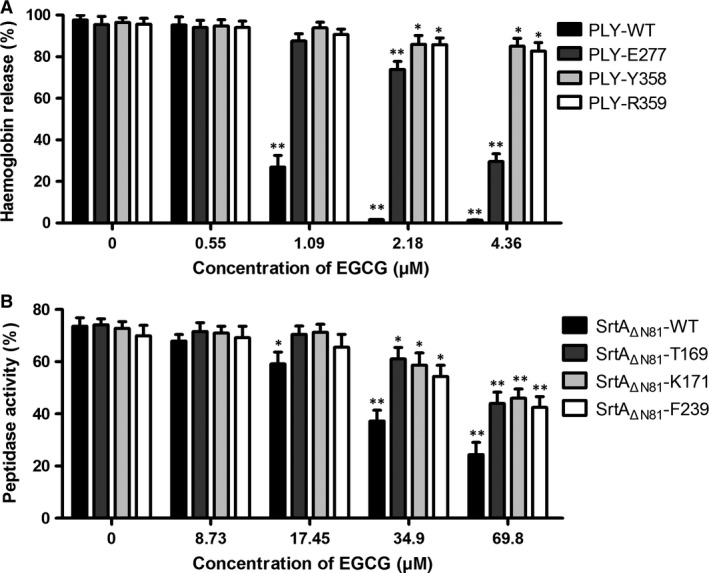
Mutation of binding sites impairs the inhibitory effects of EGCG on PLY and SrtA_ΔN81_. The inhibitory effects of various concentrations of EGCG on the haemolytic activities of purified PLY‐WT, PLY‐E277A, PLY‐Y358A and PLY‐R359A (**A**) were measured by haemolysis assays. The inhibitory effects of various concentrations of EGCG on the peptidase activities of purified SrtA_ΔN81_‐WT, SrtA_ΔN81_‐T169A, SrtA_ΔN81_‐K171A and SrtA_ΔN81_‐F239A (**B**) were measured by fluorescence resonance energy transfer (FRET) assays. The bars show the mean values of three independent assays. The error bars indicate the standard deviation (S.D.). * indicates *P* < 0.05 and ** indicates *P* < 0.01 compared with the drug‐free group according to 2‐tailed Student's *t*‐tests. EGCG: epigallocatechin gallate.

PLY variants with amino acid mutations at Glu277, Tyr358 and Arg359 were expressed and purified, and the inhibitory effects of EGCG on their haemolytic activities were assessed. The mutants did not have significantly altered haemolytic activities relative to PLY‐WT. However, EGCG did not effectively inhibit their haemolytic activities (Fig. 4A), which indicated that the mutations in the aforementioned residues impaired the interaction between EGCG and PLY. Consistent with the above results, no significant difference was observed in peptidase activity between SrtA_ΔN81_‐WT and its mutants (SrtA_ΔN81_‐T169A, SrtA_ΔN81_‐K171A and SrtA_ΔN81_‐F239A) using the FRET assay. However, EGCG did not efficiently inhibit the mutants’ peptidase activities (Fig. 4B). Thus, the interaction of EGCG with PLY at Glu277, Tyr358 and Arg359 or with SrtA at Thr169, Lys171 and Phe239 significantly attenuated PLY or SrtA biological activity.

### EGCG attenuates PLY‐mediated human alveolar epithelial cell injury

**Figure 5:**
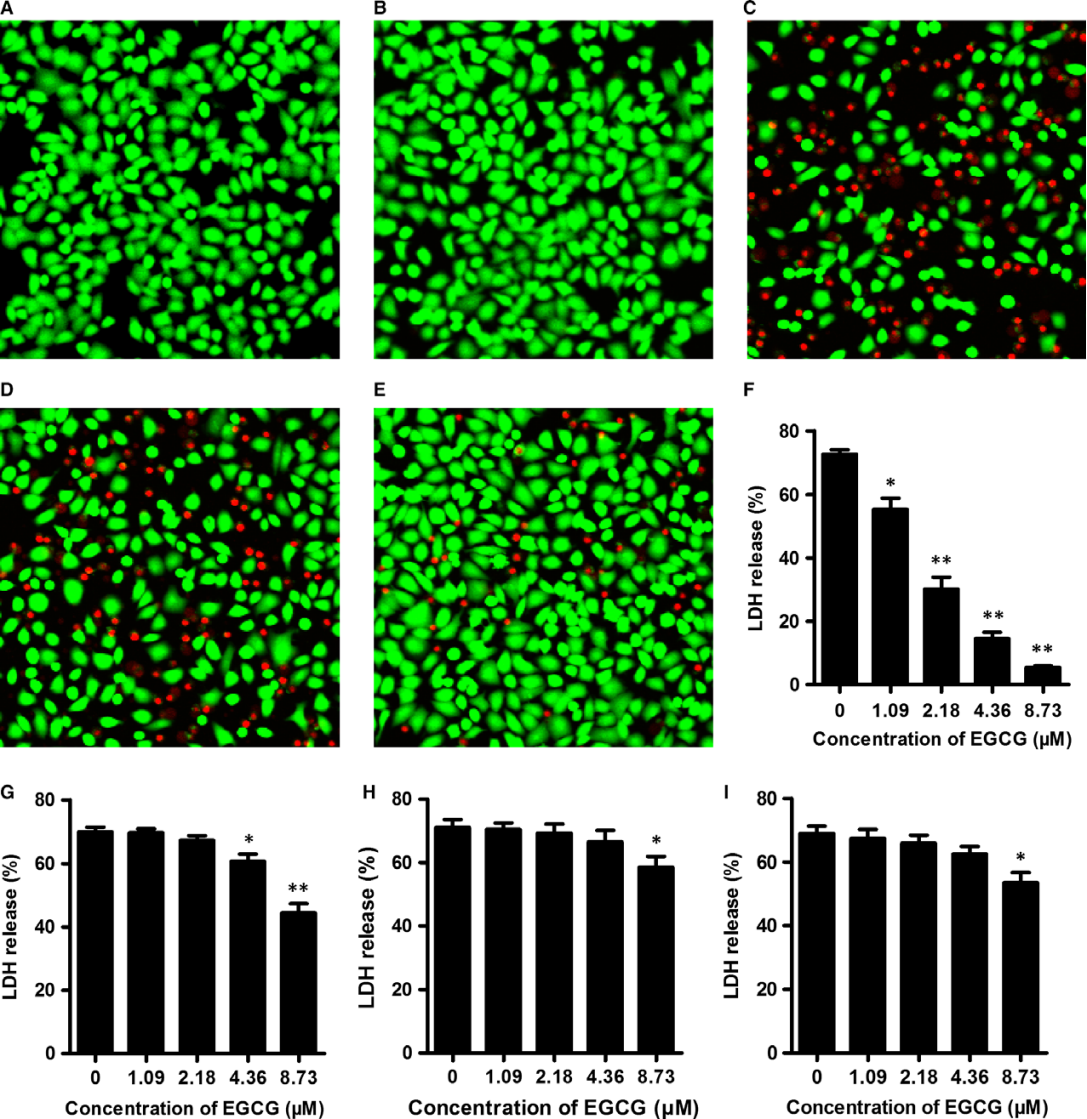
EGCG attenuates PLY‐mediated human alveolar epithelial (A549) cell injury. A549 cells were stained with a live (green)/dead (red) reagent 5 hrs after treatment with PLY, and images were captured using a confocal laser scanning microscope. (**A**) Untreated A549 cells. (**B**) Cells treated with 0.00078% (v/v) DMSO. (**C**) Cells treated with PLY‐WT in the absence of EGCG. D‐E, Cells treated with PLY‐WT pre‐incubated with 2.18 μΜ (**D**) and 8.73 μΜ (**E**) EGCG. The results shown in A–E are representative of the results from three independent experiments. LDH release by A549 cells treated with PLY‐WT (**F**), PLY‐E277A (**G**), PLY‐Y358A (**H**) or PLY‐R359A (**I**) pre‐incubated with the indicated concentrations of EGCG. The error bars indicate the S.D. * indicates *P* < 0.05 and ** indicates *P* < 0.01 compared with the drug‐free group according to 2‐tailed Student's *t*‐tests. EGCG: epigallocatechin gallate.

The pore‐forming toxin pneumolysin plays a crucial role in the pathogenesis of pneumococcal infection and is cytopathic for cultured endothelial and epithelial cells 47. We evaluated the protective effects of EGCG against PLY cytotoxicity by determining the viability of A549 cells treated with PLY pre‐incubated with various concentrations of EGCG. As shown in Figure 3, the untreated A549 cells (Fig. 5A) and the cells treated with DMSO (Fig. 5B) were stained with green fluorophores when treated with a live/dead staining reagent. The cells incubated with PLY revealed evident cell injury and death, with red fluorophores and altered cell morphology (shrunken and rounding up) (Fig. 5C). By contrast, the low concentration of EGCG (2.18 μΜ) provided moderate protection against cell injury (Fig. 5D), and 8.73 μΜ EGCG prevented the vast majority of cell death (Fig. 5E). The cell injury was quantitated by measuring LDH release, and the result was presented as the percentage of cell death. As expected, EGCG significantly inhibited PLY‐mediated A549 cell injury in a dose‐dependent manner (Fig. 5F). Additionally, the inhibitory effects of EGCG on the cytotoxicity of the PLY variants (PLY‐E277A, PLY‐Y358A and PLY‐R359A) were also assessed *via* LDH release assays. Consistent with the results of the haemolysis assay, the mutants did not have significantly altered cytotoxicity relative to PLY‐WT. However, EGCG treatment had a limited protective effect against the cytotoxicity mediated by the PLY mutants compared with the protective effect against PLY‐WT (Fig. 5G–I). Taken together, EGCG showed *in vitro* efficacy in alleviating PLY‐mediated human alveolar epithelial cell injury, and mutations in the aforementioned residues impaired the interaction between EGCG and PLY.

### EGCG inhibits *Streptococcus pneumoniae* biofilm formation

**Figure 6:**
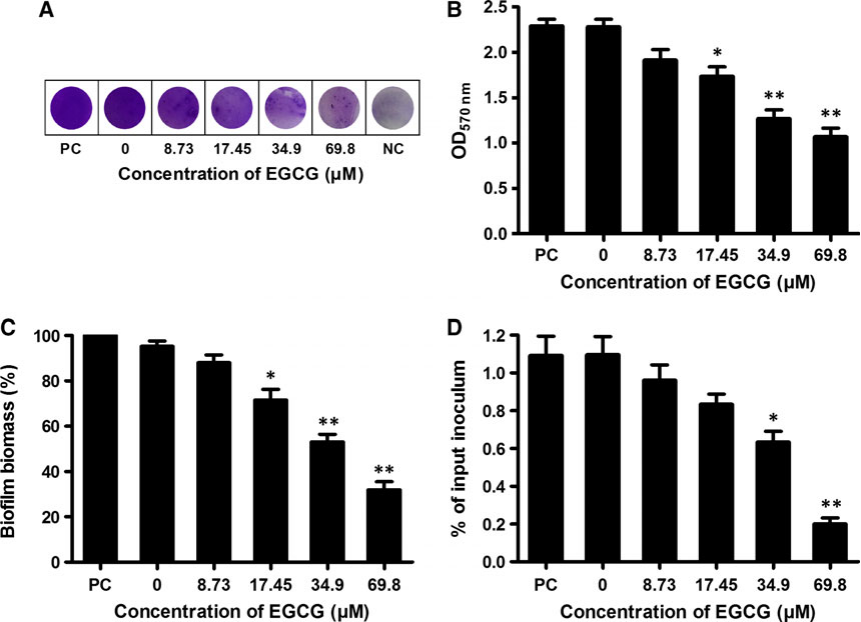
EGCG inhibits pneumococcal biofilm formation and adhesion to human epithelial cells. *Streptococcus pneumoniae* biofilms were grown in the presence of the indicated concentrations of EGCG for 18 hrs and detected by crystal violet staining and CFU counting. (**A**) Photograph of a biofilm stained with crystal violet. (**B**) Quantification of biofilm formation by measuring bound crystal violet. (**C**) Quantification of biofilm biomass by CFU counts. The biomass in the biofilm grown without EGCG was set as 100% for statistical analysis. (**D**) D39 cells pre‐cultured with the indicated concentrations of EGCG were added to Hep2 cells and then cocultured with the corresponding concentrations of EGCG for 2 hrs. After washing, D39 cells adherent to Hep2 cells were calculated by colony counting. The results shown in A are representative of the results from three independent experiments. The bars show the mean values of three independent assays. The error bars indicate the S.D. * indicates *P* < 0.05 and ** indicates *P* < 0.01 compared with the drug‐free group according to 2‐tailed Student's *t*‐tests. PC: Positive Control; NC: Negative Control. EGCG: epigallocatechin gallate.

Pneumococci form well‐organized biofilm communities during nasopharyngeal colonization and the bacteria in biofilms are highly resistant to antimicrobial agents. Previous reports have shown that SrtA inhibitor could effectively inhibit the biofilm formation of *Streptococcus mutans* 48, and neuraminidase inhibitor could reduce pneumococcal biofilm formation 15. As shown above, the peptidase activity of SrtA and the production of NanA on cell wall were effectively inhibited by EGCG (Figs 1D and 2B). We therefore examined whether EGCG could inhibit pneumococcal biofilm formation. The effect of EGCG on *S. pneumoniae* D39 biofilm formation was evaluated by crystal violet staining and biofilm biomass calculation. As expected, a significant reduction of biofilm formation was observed with increasing EGCG concentration (Fig. 6A and B), and the biofilm biomass was also significantly reduced compared to the control (Fig. 6C). We confirmed that EGCG did not influence the expression of PLY (Fig. 1B), which is correlated to some extent with biofilm formation 49, and EGCG attenuated pneumococcal biofilm formation *in vitro*.

### EGCG inhibits the adhesion of *Streptococcus pneumoniae* to human epithelial cells

As shown above, the peptidase activity of pneumococcal SrtA was significantly inhibited by EGCG. The inactivation of the *srtA* gene in *S. pneumoniae*,* Streptococcus agalactiae* and *Streptococcus gordonii* affects the localization of virulence‐associated surface proteins and decreases the adhesion of these bacteria to human epithelial cells 11, 12, 13. Additionally, the *S. pneumoniae nanA* mutant exhibits significantly reduced adhesion compared to the wild‐type strain 3, and a NanA inhibitor prevents pneumococci from adhering to pulmonary epithelial cells 20. Therefore, we examined the inhibitory effect of EGCG on pneumococcal adhesion to human epithelial cells by colony counting. Consistent with the above results, the adherence of D39 to Hep2 cells was significantly reduced following coculture with EGCG (Fig. 6D). These results indicated that EGCG may affect pneumococcal adhesion, colonization and pathogenicity *in vivo*.

### EGCG protects mice from *Streptococcus pneumoniae* pneumonia

The loss of the cytolytic properties of PLY increases the survival of mice infected with a *ply*‐deficient mutant 7, and immunization with SrtA confers protection against pneumococcal infection in mice 50. In infected mice, the *nanA*‐deficient mutant is cleared rapidly within 12 hrs from the nasopharynx, trachea and lungs 44. Because EGCG not only directly antagonized the cytolytic activity of PLY and alleviated lung cell injury but also inhibited the peptidase activity of SrtA and reduced pneumococcal adhesion to human epithelial cells *in vitro*, we further investigated whether EGCG would provide protection against *S. pneumoniae* infection in a mouse model.

**Figure 7:**
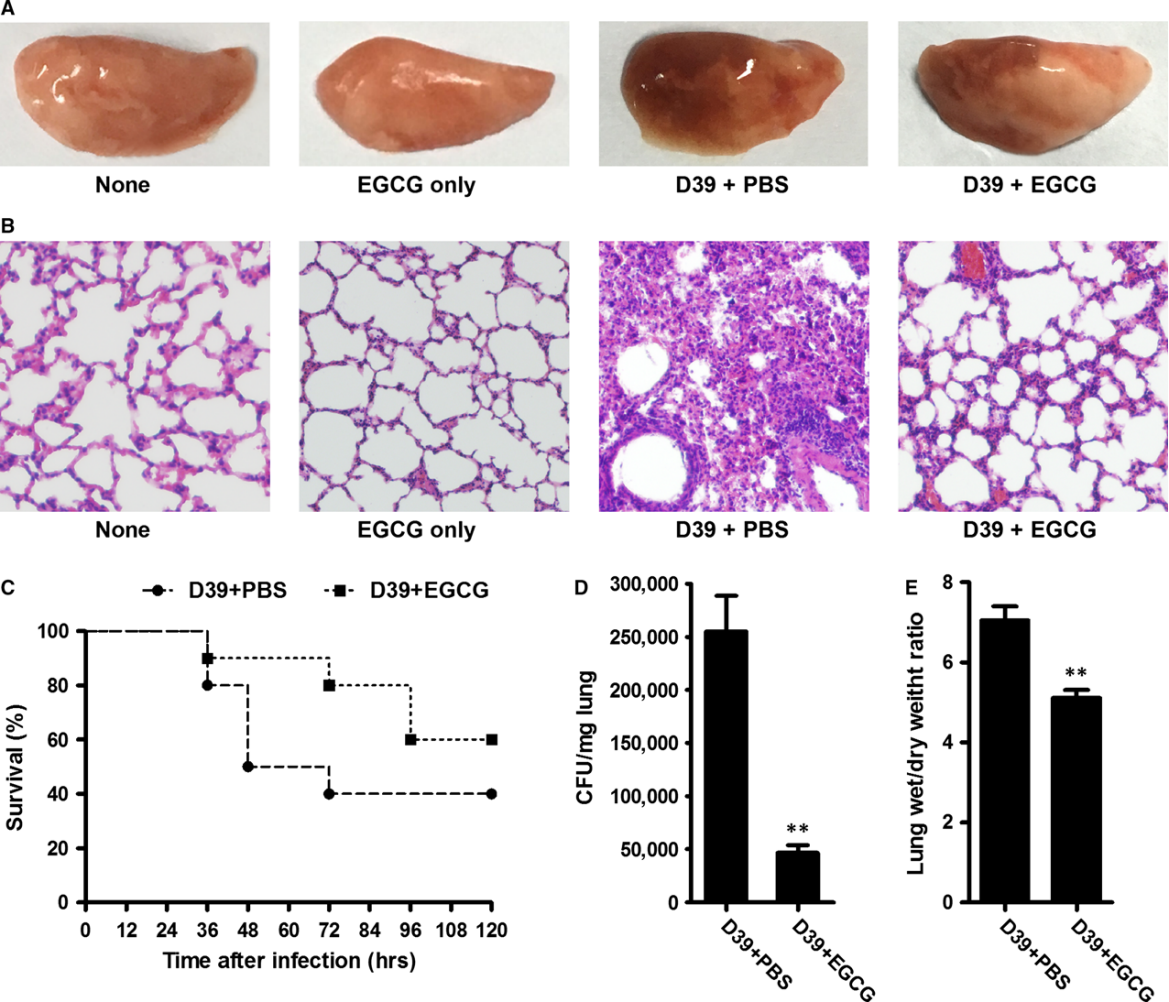
EGCG protects mice against *Streptococcus pneumoniae* pneumonia. BALB/c mice were inoculated with *S. pneumoniae via* the intranasal route and treated subcutaneously with PBS or EGCG. Each group contained 10 mice. At 48 hrs post‐inoculation, the mice were killed, the gross pathological changes (**A**) and histopathology (**B**) of lung tissue were assessed, and the bacterial burden (**D**) and wet/dry weight ratio (**E**) of lungs were calculated. Lung tissues were stained with haematoxylin and eosin (original magnification, ×100). (**C**) The mortality of mice infected with pneumococci was supervised for 120 hrs. The results shown in (**A**) and (**B**) are representative of the results from three independent experiments. The bars in (**D** and **E**) show the mean values of three independent assays. The error bars indicate the S.D. * indicates *P* < 0.05 and ** indicates *P* < 0.01 compared with the drug‐free group according to 2‐tailed Student's *t*‐tests. EGCG: epigallocatechin gallate.

Mice were intranasally inoculated with *S. pneumoniae* strain D39 following treatment with either EGCG (50 mg/kg) or PBS (containing 2% DMSO) as a control. After infection with 2 × 10^8^ CFU of pneumococci, 60% of the mice treated with PBS were killed within 72 hrs. However, the mice received EGCG exhibited a significant survival advantage, particularly at early time‐points post‐infection (Fig. 7C). The survival analysis revealed that EGCG could postpone the death of *S. pneumoniae* infected mice. The bacterial burden in the lungs was detected to assess the effect of EGCG on the colonization and survival of *S. pneumoniae* within the lungs. Bacterial survival in the lungs of infected mice treated with EGCG was significantly reduced compared with the control (Fig. 7D). Histopathological analysis of lung tissue was performed to evaluate the treatment efficacy of EGCG against pulmonary injury. Gross inspection revealed that the lungs of infected mice that received PBS were crimson and exhibited severe congestion and pulmonary oedema. By contrast, mice treated with EGCG showed light pink lungs with focal infection (Fig. 7A). Examination of the lung tissue sections (Fig. 7B) revealed significant alveolar destruction and inflammatory cell aggregation in the infected mice treated with PBS. By contrast, less destruction of the alveolar space with clear alleviation of the inflammatory reaction was observed in EGCG‐treated mice. The wet/dry weight ratio of left lung was calculated to indicate the degree of pulmonary oedema. As shown in Figure 7E, the lung wet/dry weight ratio of mice treated with EGCG was significantly decreased compared with the control. Overall, EGCG was effective in treating *S. pneumoniae* pneumonia in a murine model of infection.

## Discussion

The emergence of antibiotic‐resistant pneumococci and the poor efficacy of pneumococcal polysaccharide vaccines have complicated the treatment of *S. pneumoniae* infection 51. Undoubtedly, the abuse of antibiotics in humans and animals is the primary cause of the rapid development of drug resistance. Strategies targeting bacterial virulence rather than viability are increasingly being investigated. Numerous previous studies have demonstrated that chemical inhibitors and antibodies against virulence can significantly weaken pneumococcal virulence *in vitro* and *in vivo* 35, 52.

Pneumococcus possesses numerous virulence factors associated with pneumococcal pathogenicity, among which the pore‐forming toxin PLY is critical for pneumococcal infection and pathogenesis, including colonization in the nasopharynx and lung, transition to the cerebral spinal fluid, pulmonary injury, cardiac injury and progression of established lung fibrosis 4, 29, 53. EGCG has been reported to inhibit the intracellular growth of *L. monocytogenes* in macrophages by inhibiting the haemolytic and cholesterol‐binding activity of listeriolysin O 27. Our studies have shown that EGCG can effectively neutralize the haemolytic activity of PLY without influencing PLY expression. We further investigated the mechanism by which EGCG antagonizes PLY‐mediated haemolytic activity *via* an oligomerization assay, MD simulations and mutational analysis. Our results suggested that the interaction of EGCG with PLY residues (Glu277, Tyr358 and Arg359) reduces the oligomerization of PLY monomers, a critical step for pore formation, thus inhibiting PLY‐mediated haemolytic activity. The PLY mutants (PLY‐E277A, PLY‐Y358A and PLY‐R359A) were much less sensitive to EGCG, as evidenced by the lower inhibitory effect of EGCG against PLY‐mediated haemolytic activity and cytotoxicity compared with PLY‐WT.

In addition, we identified EGCG as an effective inhibitor of SrtA, another important virulence factor in *S. pneumoniae*. In Gram‐positive bacteria, SrtA is closely associated with the anchoring of surface proteins 11. The inactivation of *srtA* gene significantly attenuates the display of surface proteins and bacterial virulence 11, 12, 13. Pneumococcal NanA, the most investigated surface protein anchored by SrtA through the LPETG motif, is essential for bacterial colonization and biofilm formation. Pneumococci in biofilms exhibit increased resistance to antimicrobial agents, which is partly attributable to the lower penetration of antibiotics into the biofilm structure. Therefore, the biofilm functions as a shield to protect the bacteria from antimicrobials 18. As expected, inhibition of SrtA activity by EGCG significantly inhibited the production of NanA on the pneumococcal cell surface. The interaction between EGCG and SrtA was further confirmed by MD simulations and mutational analysis. Our results suggested that the interaction of EGCG with the active‐site residues Thr169, Lys171 and Phe239 reduces SrtA biological activity. Furthermore, when NanA is not localized normally, pneumococcal biofilm formation and bacterial adhesion are seriously affected. As expected, when *S. pneumoniae* D39 was cocultured with EGCG, biofilm formation and adherence to human epithelial cells were significantly attenuated. In agreement with the effects of EGCG against PLY, the activity of the SrtA mutants (SrtA_ΔN81_‐T169A, SrtA_ΔN81_‐K171A and SrtA_ΔN81_‐F239A) was similar to that of SrtA‐WT; however, the inhibitory effect of EGCG against the SrtA mutants (SrtA_ΔN81_‐T169A, SrtA_ΔN81_‐K171A and SrtA_ΔN81_‐F239A) was much lower than that against SrtA‐WT. Furthermore, treatment with EGCG provided protection against *S. pneumoniae* pneumonia in mice and reduced the pathological injury and bacterial burden in the lungs. Taken together, these results suggest that EGCG could be a potential antivirulence agent for *S. pneumoniae* infection without bactericidal activity. These excellent medicinal values of EGCG further support the benefits of green tea for human health and lay the foundation for further study of antivirulence strategies.

## Conflict of interest

The authors have no conflict of interest to declare.
